# Encoded Microspheres in Multiplex Detection of Mycotoxins and Other Analytes

**DOI:** 10.3390/foods15020247

**Published:** 2026-01-09

**Authors:** Wenhan Yu, Haili Zhong, Xianshu Fu, Lingling Zhang, Mingzhou Zhang, Xiaoping Yu, Zihong Ye

**Affiliations:** 1 Key Laboratory of Microbiological Metrology, Measurement & Bio-Product Quality Security, State Administration for Market Regulation, College of Life Sciences, China Jiliang University, Hangzhou 310018, China; ywh1835628626@163.com (W.Y.); p24091055087@cjlu.edu.cn (H.Z.); zmzcjlu@cjlu.edu.cn (M.Z.); yxp@cjlu.edu.cn (X.Y.); zhye@cjlu.edu.cn (Z.Y.); 2 Linping District Center for Disease Control and Prevention of Hangzhou City, Hangzhou 311199, China; tianqin1984@163.com

**Keywords:** encoded microspheres, flow cytometry, mycotoxin detection, suspension array technology

## Abstract

This paper provides a systematic review of the progress in encoded microsphere suspension array technology and its application in the multiplex detection of mycotoxins. Mycotoxins are diverse and frequently coexist in food matrices, leading to synergistic toxic effects. This poses significant challenges to existing risk assessment systems. Current multiplex detection methods still face technical bottlenecks such as target loss, matrix interference, and reliance on large-scale instruments. Suspension array technology based on encoded microspheres, combined with efficient signal amplification strategies, offers an ideal platform for achieving highly sensitive and high-throughput analysis of mycotoxins. This paper systematically reviews the core aspects of this technology, including encoding strategies such as physical, optical, and multi-dimensional approaches, along with new encoding materials like aggregation-induced emission materials and fluorescent proteins. It further covers matrix materials and preparation methods with an emphasis on green, biocompatible options and integrated fabrication techniques, as well as signal amplification mechanisms based on nucleic acid amplification, enzyme catalysis, and nanomaterials. The integration of magnetic separation techniques and the combination with portable, smartphone-based platforms for intelligent on-site detection are also highlighted. Finally, this review outlines future development trends such as the incorporation of artificial intelligence, 3D printing, and smart algorithms, aiming to provide theoretical references and technical support for research and applications in related fields.

## 1. Introduction

Mycotoxins exhibit remarkable diversity, with over 400 known species identified to date [1,2]. Multiple toxins frequently coexist within the same food matrix, triggering synergistic toxic effects [3,4,5,6] that pose significant challenges to existing risk assessment systems. However, achieving simultaneous detection of multiple toxins remains constrained by several technical bottlenecks. First, owing to the significant differences in the chemical structure, polarity, and solubility of various toxins, commonly used sorbents exhibit distinct adsorption selectivity, which can easily lead to the loss of target analytes [7]. Secondly, components in complex food matrices, such as cereal residues, lipids, pigments, and proteins, significantly interfere with optical or electrochemical signal readings during trace detection [8]. This interference forces traditional methods to rely on cumbersome pretreatment steps to obtain accurate results. Finally, although some detection methods are highly sensitive, they still depend heavily on large-scale instruments, making them unsuitable for rapid on-site screening. The development of suspension biochip technology centered on multiplexed encoded microspheres, combined with integrated efficient signal amplification strategies, is expected to overcome these technical barriers. This approach could enable ultra-sensitive, high-throughput analysis of mycotoxins, providing critical technological support for systematically evaluating their combined toxicity and ensuring food safety.

In this context, the xMAP (Multi-Analyte Profiling) technology launched by Luminex in 1997, also referred to as suspension array technology, represented a major breakthrough by enabling parallel detection of multiple targets instead of just a single target [9]. Subsequently, suspension array technology using encoded microspheres has become one of the most promising multiplex detection platforms, thanks to its fast reaction kinetics, flexible array design, and excellent multiplex analysis performance [10,11,12]. Compared with traditional solid-state biochips, this technology demonstrates advantages at multiple levels. Its core strength lies in the use of diverse encoding strategies for microspheres, which enable simultaneous detection of multiple targets and greatly increase detection throughput [13,14]. At the same time, microspheres offer a high specific surface area, strong loading capacity, controllable morphology, and easily modifiable surface properties, which allows them to meet high-throughput and diversified detection needs [15,16,17]. Furthermore, due to its unique liquid-phase reaction system, the technology provides conditions that are closer to a biological environment. This not only helps maintain the stability of biomolecules but also significantly speeds up molecular recognition and binding, thus enhancing detection efficiency and reliability [18,19,20]. With technological advancements and expanding applications, the global encoded microsphere market continues to grow. Companies such as Becton Dickinson and Thermo Fisher Scientific are promoting industrialization by offering standardized magnetic bead-fluorescent encoding solutions like MagPlex^®^, highlighting the field’s promising future.

This review provides a systematic introduction to liquid-phase suspension biochip technology based on encoded microspheres, with a focus on recent advances in physical and optical encoding strategies, matrix material selection, and microsphere preparation methods. It compares the physicochemical properties and synthesis approaches of various encoding elements, discusses current challenges and potential improvements, and summarizes signal amplification strategies based on nucleic acid amplification, enzyme catalysis, and nanomaterials. The application potential of this technology in multiplex mycotoxin detection is also thoroughly explored. While earlier reviews have addressed suspension arrays or mycotoxin detection methods, they often do not systematically relate recent progress in encoded microsphere technology to the practical needs of multiplex mycotoxin analysis, especially in handling complex samples, achieving high sensitivity, and enabling point-of-care detection. To bridge this gap, this review offers an integrated perspective on how encoded microsphere-based platforms can overcome the limitations of conventional detection approaches. It emphasizes innovations in encoding strategies, eco-friendly and controllable fabrication of matrix materials, and the incorporation of efficient signal amplification mechanisms. Looking forward, it also outlines the transition toward intelligent and portable detection systems, thereby providing a coherent theoretical framework and technical roadmap to guide future research and applications in this evolving field.

## 2. Encoding Strategies for Microspheres

Liquid-phase suspension biochip technology is centered on encoded microspheres, which use specific probes attached to their surfaces to capture and recognize target molecules [21,22]. In this system, the barcode of each microsphere is used to distinguish different detection targets, while reporter signals allow for quantitative analysis. Developing high-capacity encoding strategies and matching decoding technologies is essential for improving the ability to test for multiple targets at once. These encoding strategies can be based on the microsphere’s size [23], shape [24,25], magnetic properties [26,27], or optical features [28,29,30,31,32], or can combine these elements [33,34]. Currently, decoding is no longer limited to traditional flow cytometers. It now also uses other systems like fluorescence microscopes, micro-resistance counting platforms, and smartphones. This expansion meets the increasing need for high-throughput and portable detection devices.

### 2.1. Physical Encoding

**Figure 1:**
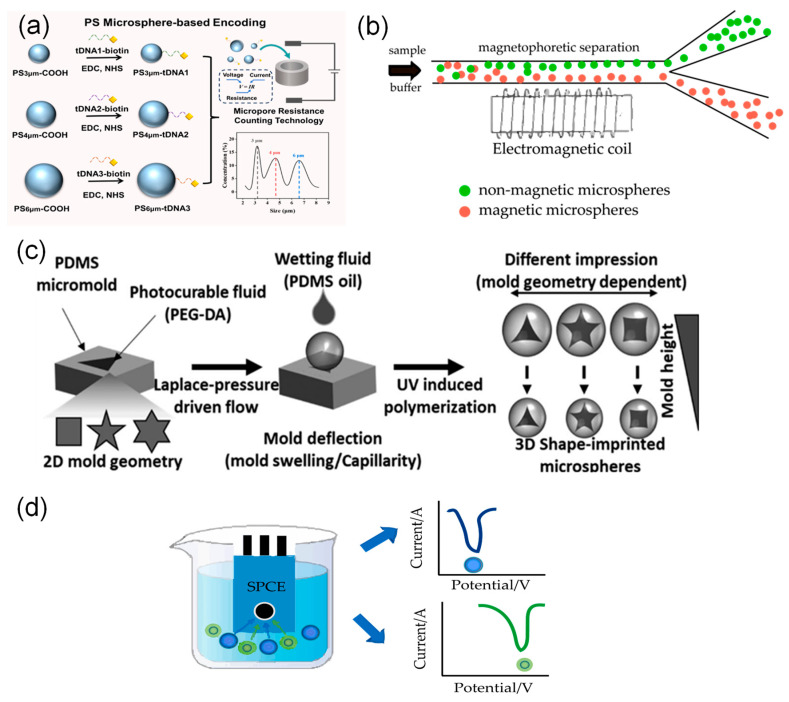
Physical encoding strategies. (**a**) Size encoding: multiplexed detection via size encoding of polystyrene microspheres and resistive pulse counting [23]. (**b**) Magnetic encoding: A microfluidic detection system based on magnetic encoding achieves target separation via magnetic enrichment. (**c**) Shape encoding: By utilizing shape-encoded microspheres with fluorescence microscopy and image analysis software, allowing for target differentiation [35]. (**d**) Electrochemical encoding: By magnetically enriching the encoded microspheres on a screen-printed carbon electrode (SPCE) and reading the characteristic electrochemical signals through square wave voltammetry (SWV) scanning, simultaneous quantification of the two targets is achieved.

Physical encoding strategies for microspheres create unique barcodes by altering key physical features such as size, magnetic properties, shape, and electrochemical traits. This method makes it possible to distinguish different targets in high-throughput detection. The following section describes common strategies (Figure 1) and their key characteristics.

Size-based encoding uses microspheres of different sizes as barcodes to identify different targets. The resulting signals can be detected by instruments such as flow cytometers or micro-resistance counting platforms [23,36,37]. For example, Li et al. [23] attached different target DNA strands to polystyrene microspheres of distinct sizes. They then used microchannel resistive counting to identify characteristic peaks corresponding to each microsphere size, enabling simultaneous detection of multiple targets (Figure 1a).

Size-based encoding may affect quantitative analysis because microspheres of different sizes react at different rates. Additionally, flow cytometers have a limited ability to distinguish between similar sizes [38]. To overcome these issues, Sheng et al. [27] first proposed a suspension array based on magnetically encoded plasmonic microspheres. Three types of plasmonic microspheres with magnetic contents of 5.0%, 1.6%, and 0.5% were selected to specifically recognize different target molecules. The magnetic force applied to the microspheres was progressively enhanced by increasing the current intensity. When the magnetic force became sufficiently strong, the microspheres detached from the bottom and migrated into another chamber, thereby achieving decoding and classification of the mixed microspheres. In a further development, Hong et al. [39] created a microfluidic detection system based on dual magnetic and size-encoded microspheres, employing magnetic (5 μm) and non-magnetic microspheres (5 μm, 10 μm) to capture H7N3, H1N1, and H3N2 viruses, respectively. The system achieves target separation and enrichment through a custom microfluidic chip (incorporating a magnetic separation zone and a wedge-shaped size detection zone). It utilizes quantum dot fluorescence intensity for online detection of viral subtype concentrations, thereby enabling highly sensitive detection of multiple pathogens. (Figure 1b).

Advances in microfluidics and flow lithography have established shape encoding as an effective approach for high-throughput multiplex detection. Zhao et al. [40] developed a multiplex miRNA detection platform based on shape-encoded hydrogel microspheres integrated with a microfluidic chip for rapid, high-throughput analysis. In their method, pattern arrays of crosses and circles were designed using software such as AUTOCAD and fabricated into high-resolution photomasks. In this process, UV light was shone through the templates onto the probe-containing mixture, instantly solidifying it into many shape-defined microspheres. Consequently, each microsphere shape was functionally encoded to specifically capture its corresponding target miRNA. Finally, multiplex detection was accomplished by analyzing the fluorescence images of the microspheres. In a similar study, Ganguly et al. [35] developed a straightforward microforming process based on the interfacial deformation of a photocurable fluid to create functionalized microspheres. In this technique, the shape control was achieved by using poly (dimethylsiloxane) micromolds with different two-dimensional geometries, such as triangle, square, and star patterns; upon photopolymerization, these molds transferred their precise negative imprints onto the microsphere surfaces. Concurrently, the size of the microspheres could be tuned independently by simply varying the depth of the molds, which directly determined the volume of the photocurable fluid and thus the final particle diameter. The researchers then conjugated different antibodies to microspheres of specific shapes and incubated them with fluorescently labeled secondary antibodies. The encoding signals, based on the distinct shapes, were acquired using an inverted fluorescence microscope and interpreted with image analysis software, highlighting the applicability of this shape-encoded system in rapid biological detection (Figure 1c).

Despite these advances, physical encoding strategies still face challenges including limited encoding capacity and insufficient signal resolution. Size-based encoding depends on differences in light scattering, but its ability to distinguish between microspheres decreases when the particle size range is narrow. Magnetic encoding faces difficulties in high-throughput decoding, and microspheres with complex shapes raise concerns about fabrication and biocompatibility. To address these limitations, Hu et al. [41] developed electrochemically encoded microspheres. They used the characteristic potentials of redox-active molecules to create unique “electrochemical fingerprints” and measured the ratio of peak potential to current with an electrochemical workstation. This method increases encoding capacity while also allowing for target identification and quantification (Figure 1d).

### 2.2. Optical Encoding

**Figure 2:**
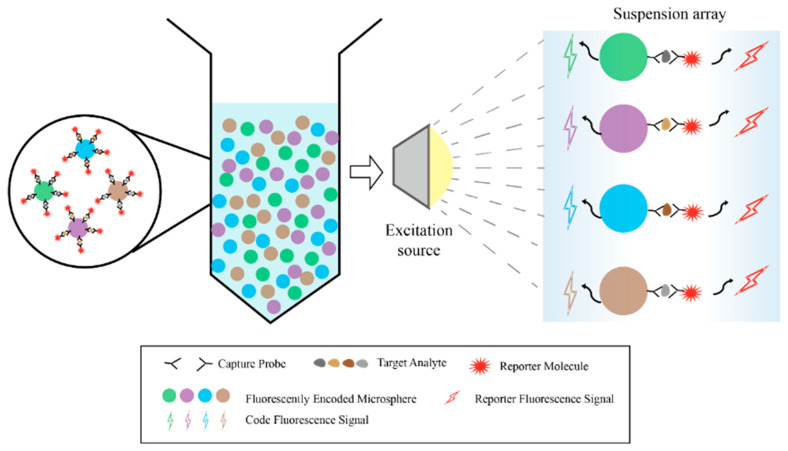
Schematic diagram of a typical optical-encoded suspension array platform.

In practice, physical encoding has several limits. Its capacity is usually under 10 types, and its detection limit is often in the ng/mL range [23,35,39]. Also, because image-based physical encoding must scan each view one by one, its processing speed is low. In contrast, optical encoding is now the most common method. It can encode tens to hundreds of targets and has a much lower detection limit, down to pg/mL [22,28]. When used with high-throughput tools like flow cytometry, it can detect thousands to tens of thousands of particles per second, much faster than physical encoding. Optically encoded microspheres are also easier to make, cost less, and give clearer signals [42,43,44,45]. This technology builds barcode libraries using microspheres with different colors. Each microsphere surface is coated with a specific capture probe. After capturing the target analyte, detection reagents with fluorescent reporter molecules are added for identification. This finally forms a composite structure made up of the microsphere, capture probe, target analyte, and reporter molecule. During detection, a flow cytometer uses lasers to excite each microsphere as it passes through the detection zone. It reads both the encoded signals for identification and the reporter signals for quantitative analysis, enabling synchronous and quantitative detection of multiple target molecules in the sample (Figure 2). Fluorescent encoding elements mainly include organic dyes, quantum dots, upconversion nanoparticles, SERS tags, and fluorescent proteins. Ideal encoding materials should have large Stokes shifts, narrow emission peaks, and show good stability and biocompatibility. For encoding strategies, building high-capacity barcode libraries to support high-throughput, multiplexed detection can be done in several ways. These approaches include precisely adjusting fluorescence wavelengths and intensities, designing optical structures, utilizing fluorescence lifetime differences, or planning spatial distribution. These strategies thereby have distinct focuses regarding key performance metrics across different optical dimensions, as compared in Table 1.

#### 2.2.1. Encoding Strategies Based on Wavelength and Intensity Spectral Characteristics

Organic fluorescent dyes are still among the most widely used materials in optical encoding [47,48]. As an example, Luminex xMAP technology uses red and orange dyes embedded in microspheres at different concentration ratios, combined with a green reporter dye to measure target analytes [9]. However, these dyes have certain limitations, such as limited encoding diversity, susceptibility to fluorescence quenching and photobleaching, and the need for multiple excitation wavelengths, all of which can restrict their performance and applications [28,49]. In recent years, novel fluorescent materials like quantum dots, upconversion nanoparticles, and SERS nanoparticles have gradually overcome the limitations of organic dyes by offering superior optical properties and encoding capabilities. As a result, these materials show broad potential for use in multiple fields such as biosensing, materials science, and information storage [97,98,99].

Quantum dots (QDs) are nanoscale fluorescent materials that emit light from the visible to near-infrared range when excited [100]. Compared to other fluorescent materials, quantum dots have unique optical properties that make them more suitable for spectral encoding [50,51,52,53,54,55,56,57]. Since the concept of quantum dot spectral encoding was introduced by Nie’s team in 2001 [43], encoding strategies using different colors and fluorescence intensities have been widely used for multiplex detection. Theoretically, the number of possible barcodes can be calculated using the formula $C\;=\;M^{N}\;-\;1$ where M is the number of intensity levels and N is the number of colors [101]. Zhang et al. [102] created 30 different barcodes using two types of quantum dots at six intensity levels, allowing for the highly sensitive detection of four tumor markers by flow cytometry. However, the number of codes that can be used in practice is usually lower than the theoretical maximum. This is mainly due to effects such as photon reabsorption and fluorescence resonance energy transfer (FRET) between different quantum dots, which cause spectral interference and make barcodes hard to distinguish [58,59,60]. One way to reduce this energy transfer is to use quantum dots with large Stokes shifts, which increases the separation between excitation and emission wavelengths [103,104,105]. For example, Wu et al. [22] built a dual-color encoding system with seven intensity levels using four-legged CdSe/CdS quantum dots, which allowed for the effective detection of five allergens. In another approach, Zhang et al. [106] designed host-guest structured microspheres to physically separate different fluorescent labels. They placed quantum dots inside a central host core and attached dye-modified guest particles on the surface, keeping them more than 10 nm apart, which is beyond the typical range of FRET, thus avoiding optical interference in multicolor barcodes. Other methods such as quantum dot nanobeads and silica coating have also been used to improve the dispersion and stability of quantum dots [107,108]. Lv et al. [109] used polyethylene glycol to modify entangled quantum dots and then applied a silica coating, which greatly improved their stability. Besides causing spectral overlap in encoding, FRET can also increase background fluorescence, which may interfere with reporter signals and reduce sensitivity for detecting low-concentration targets. Therefore, it is helpful to use different emission bands for encoding and reporter signals, or to physically separate them using core–shell structures, to ensure accurate detection [60]. Biosafety is another important factor for real-world applications. Traditional II–VI quantum dots often contain heavy metals such as cadmium or lead, which limits their use in biological systems. In recent years, newer materials such as lower-toxicity I–III–VI quantum dots (like CuInS_2_), carbon dots, and graphene quantum dots have shown promise as encoding elements. These offer good biocompatibility, tunable light emission, and lower environmental impact [61,62,63,64,110,111,112,113].

In suspension arrays that use organic dyes or quantum dots, short-wavelength excitation light often causes strong Rayleigh scattering and autofluorescence in biological samples, leading to high background signals [114]. In comparison, near-infrared light produces very low background fluorescence. Upconversion nanoparticles, which are lanthanide-doped nanocrystals, can be excited by near-infrared light and then emit higher-energy short-wavelength light [68,115,116]. These nanoparticles can also be optically encoded by varying their fluorescence color and intensity. Common approaches to adjust their properties include changing the type and concentration of dopant ions [117,118,119], using FRET effects through surface modifications [120,121,122], adding metal components to increase luminescence [123,124], or building core–shell structures to reduce surface quenching [125]. However, most commercial flow cytometers are equipped with UV or visible lasers and do not have near-infrared excitation modules needed for upconversion nanoparticles, which has limited their widespread use. To address this, Zheng et al. [72] used Er/Tm-doped upconversion nanoparticles to create green and blue emitting microspheres and developed a custom microfluidic platform integrated with near-infrared optical tweezers. This system allowed for stable and automated detection of upconversion luminescence, offering a practical solution for using these nanoparticles in encoded analysis.

Aggregation-induced emission materials (AIEgens) are a new type of luminescent molecules that show much stronger fluorescence in aggregated or solid states. This property effectively solves the aggregation-caused quenching problem seen in traditional materials [126,127,128,129]. Besides this unique feature, AIEgens also offer benefits such as tunable emission wavelengths, low background interference, and high signal intensity, making them well-suited for building high-performance encoded microspheres [75,76,77]. Encoding methods using AIEgens mainly involve changing the emission wavelength by adjusting the molecular structure, along with using fluorescence intensity to create multi-dimensional barcodes. Wu et al. [73] first developed a suspension array using four types of AIEgens, creating 30 distinguishable barcodes to detect five allergens at the same time. This method showed sensitivity three times higher than quantum dot systems and five times higher than organic dye systems. Due to the broad emission spectra of AIEgens, researchers often combine their single-wavelength emission with intensity-based or size-based encoding [64,78].

Fluorescent proteins are natural or engineered proteins capable of absorbing light at specific wavelengths and emitting light at longer wavelengths. Thanks to their excellent optical properties and high biocompatibility, these proteins have shown significant value in applications like cell imaging, tumor visualization, and biosensing [130,131]. As encoding elements, fluorescent proteins can be used to build barcode libraries by adjusting their types and expression levels. They offer advantages such as large encoding capacity, high signal-to-noise ratio, and good biocompatibility [28]. However, research in this area is still limited. In a recent study, Zheng et al. [28] developed an encoding system with 506 distinguishable barcodes by varying the types and concentrations of three fluorescent proteins, mTagBFP, EGFP, and DsRed, and achieved high-capacity encoding for multiplex nucleic acid detection.

#### 2.2.2. Encoding Strategies Based on Optical Structures

Surface-enhanced Raman scattering (SERS) encoding relies on the enhancement of Raman signals. While Raman spectroscopy can reflect molecular vibrational and rotational information, its intrinsic signal is very weak. When noble metal nanostructures such as gold or silver are introduced, localized surface plasmon resonance is excited, which greatly amplifies the Raman signals, creating the SERS effect [132,133,134]. SERS-encoded microspheres are usually prepared by attaching or encapsulating specific Raman reporter molecules onto their surfaces, and distinct spectral barcodes are generated based on differences in the wavelength and intensity of these molecules [135,136,137,138,139]. As an example, Jun et al. [140] assembled silver nanoparticles on sulfonated polystyrene magnetic beads and adsorbed three different Raman reporter molecules to form distinguishable spectral tags, which were used for protein-specific recognition. Clearly, SERS encoding can effectively address the issues of spectral overlap and signal attenuation often seen in traditional fluorescence methods [81,82,83]. However, several key challenges remain for practical use of this technology. First, the uneven distribution of SERS “hotspots” on microsphere surfaces results in poor signal reproducibility [85,86]. To tackle this, Zou et al. [80] used droplet-based optofluidic technology to fabricate encoded magnetic beads with periodic SERS arrays. The resulting stable “focal beacons” significantly improved detection reliability. Second, metal nanoparticles often bind unstably to Raman reporter molecules, and Raman signals are susceptible to crosstalk from fluorescent labels. Solving this requires careful selection of Raman dyes and optimized labeling strategies [87]. Moreover, SERS systems usually depend on expensive instruments, which has limited their broader adoption. A proposed low-cost autoencoder strategy based on a confocal Raman microscope by the Gao team offers a new direction for promoting wider use of SERS technology [79].

Photonic crystals are another important medium for optical structural encoding. They control the movement of photons through periodic dielectric structures [89]. The color shown by photonic crystal-encoded microspheres comes from their internal physical structure, which avoids problems such as photobleaching or fluorescence quenching. These microspheres can also reflect specific colors under ordinary white light during decoding, making complex excitation equipment unnecessary [141]. In one study, Zhao et al. [88] combined photonic crystal barcodes with a sandwich immunoassay to simultaneously detect two targets: the SARS-CoV-2 spike protein and nucleocapsid protein. The detection limits were 0.478 ng/mL and 0.255 ng/mL, respectively, showing high sensitivity, accuracy, and specificity.

#### 2.2.3. Encoding Strategies Based on Temporal and Spatial Characteristics

Beyond traditional encoding based on fluorescence color and intensity, monochromatic signal channel techniques now also make use of additional dimensions like fluorescence lifetime [34,60,91,93,142,143,144] and anisotropy [94].

Fluorescence lifetime refers to the average time a fluorescent molecule remains in an excited state before returning to the ground state. This parameter can be used for encoding and is independent of absolute intensity [34]. Advances in time-resolved instruments have made it easier to decode microspheres encoded by fluorescence lifetime. Interestingly, the FRET effect can be beneficial in lifetime encoding, as adjusting the distance between the donor and acceptor allows for precise control of the fluorescence lifetime; shorter distances produce shorter lifetimes [60,92]. Chen et al. [60] developed four FRET-based lifetime-encoded systems by using energy transfer between lanthanide ions (Tb^3+^/Eu^3+^) and quantum dots. They achieved clear signal discrimination through time-gated photoluminescence intensity detection. In a similar way, varying the concentration of doped ions in upconversion nanomaterials can also produce encoded particles with distinct lifetimes [34,93].

Fluorescence anisotropy (FA) can distinguish spectrally overlapping fluorescence signals by using differences in emission from different polarization directions. This approach also helps to overcome problems caused by spectral crosstalk [94]. However, FA measurements can be affected by molecular interactions and environmental interference [95,96]. To address this, Huang et al. [94] designed a DNA nanostructure similar to a peptide scaffold, which allowed for the precise placement of fluorophores at specific sites within the DNA framework. This design effectively improved the stability of anisotropy signals against interference. Furthermore, by carefully controlling the spatial positions of fluorescent molecules on the DNA framework, the researchers created eight stable barcodes. This work offers a new method for highly stable multiplex encoding.

### 2.3. Combining Multiple Encoding Methods Increases Encoding Capacity

To increase encoding capacity, researchers often combine different encoding elements to perform multidimensional encoding. Flow cytometry can analyze fluorescence signals, microsphere size, and refractive index at the same time, providing a technical basis for this approach [36,145]. Wang et al. [33] used a dual-encoding method for polystyrene microspheres that combined fluorescence spectra with variations in microsphere size. Each barcode represented a specific pathogen. With the support of computer vision algorithms that automatically analyze color, size, and quantity, they achieved simultaneous detection of four pathogens. At the same time, encoding strategies that combine spectral and fluorescence lifetime properties have also received much attention. Zhou et al. [34] achieved independent control over emission wavelength and lifetime by adjusting the dopant type and the thickness of the energy relay layer in upconversion nanoparticles. Their proposed wavelength/lifetime binary encoding method increased the encoding capacity by three orders of magnitude compared to traditional wavelength/intensity strategies. Based on this work, they also developed a time-resolved imaging system capable of analyzing both wavelength and lifetime information simultaneously.

## 3. Construction of Encoded Microspheres: Matrix Materials and Preparation Methods

The performance of encoded microspheres, including the stability of encoded signals, biocompatibility, and suitability for multiplexed detection, mainly depends on the physicochemical properties of the matrix materials and how they are prepared. The matrix material affects the environment in which encoding elements are loaded, the ability to functionalize the surface, and the overall stability of the microspheres. At the same time, the preparation method influences how uniformly encoding elements are distributed, the monodispersity of the microspheres, and the reproducibility of the manufacturing process [49,146,147]. Therefore, the close relationship between these two factors is essential for producing high-performance encoded microspheres. Currently, there are two main approaches for incorporating fluorescent dyes into microspheres: surface modification and matrix integration. Surface modification fixes dyes through covalent coupling, physical adsorption, or other non-covalent interactions, while matrix integration embeds dyes inside the microspheres during their formation. Table 2 systematically compares the technical principles and characteristics of different fabrication strategies.

### 3.1. Surface Modification Strategies

**Figure 3:**
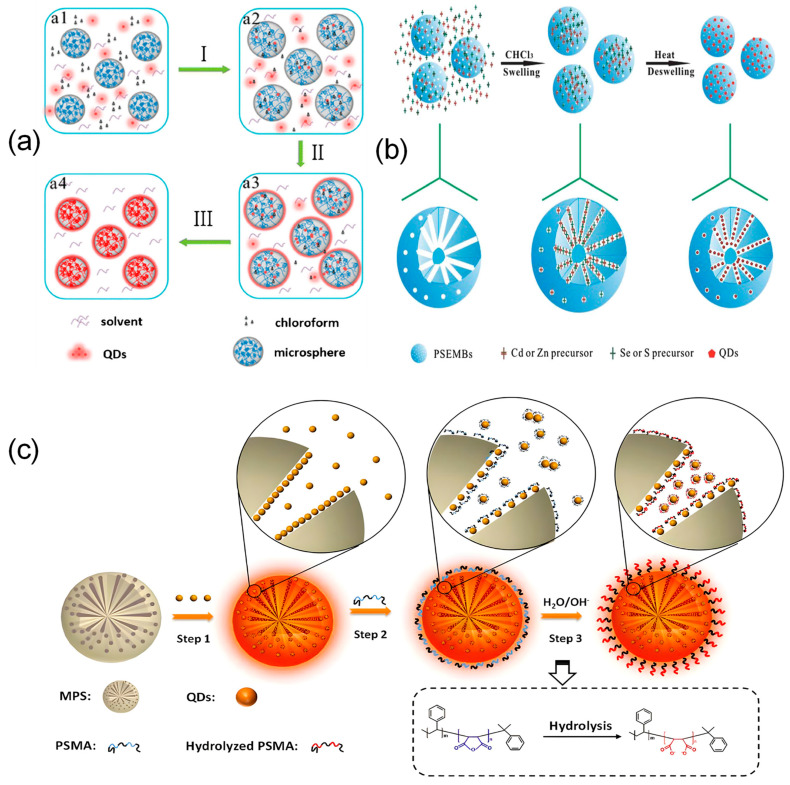
Technological development process of the swelling method. (**a**) Swelling-shrinking process: cooling evaporation method, where steps I, II, and III, respectively, show nanoparticle permeation into microspheres, heating-induced swelling, and cooling-induced shrinkage [169]. (**b**) One-step optimization method encapsulating in situ synthesized QDs within microspheres [149]. (**c**) Surface functionalization. Step 1: Adsorption, loading quantum dots into mesoporous microspheres; Step 2: Adding PSMA polymer to the mixture; Step 3: Adding NaOH aqueous solution triggers hydrolysis of the anhydride groups on the PSMA chain, converting them into hydrophilic carboxyl groups (-COOH) [171].

Surface modification strategies mainly use pre-synthesized microspheres as the base for encoding. Silicon dioxide [139,159,160] and polystyrene [23,32,161,162,163] are currently the most widely used support materials for encoded microspheres. Silica microspheres offer high specific surface area, good thermal stability, and abundant surface silanol groups that support functionalization [164]. Polystyrene microspheres are strongly hydrophobic, low in cost, and easy to process [165]. These properties make both materials ideal substrates for surface modification. Besides these common supports, other polymer matrices such as polycaprolactone microspheres (PCL) [166], polyphosphazene microspheres (FPZS) [138], and glycidyl methacrylate microspheres (APGMA) [167] can also be used as matrix materials for fluorescent encoding. For such pre-synthesized microspheres, the swelling method and the layer-by-layer self-assembly (LBL) are classical and well-established preparation approaches. The basic principle of the swelling method involves using organic solvents to expand porous microspheres, allowing fluorescent materials to enter. When the solvent is removed by exchange or evaporation, the microspheres shrink and trap the fluorescent materials inside. Hydrophobic and hydrophilic interactions, concentration gradients, and electrostatic forces are the main drivers that help move fluorescent materials into the microspheres [30]. The swelling method has evolved through three main stages: the swelling-shrinking process, one-step optimization, and surface functionalization, as shown in Figure 3. In early research, the swelling method mainly used concentration gradients, hydrophobic interactions, or charge interactions to draw quantum dots into microspheres [43,168]. Later, Song [169] added a cooling-induced shrinking step to improve quantum dot loading efficiency (Figure 3a). However, polar solvents often cause fluorescence quenching [148]. To address this, the We team [170] developed a toluene-swelling and hexane-shrinking method that worked effectively. The resulting all-organic solvent-based microspheres maintained long-term fluorescence stability in water. In contrast, Yang et al. [149] developed a one-step method that avoided multiple reaction steps. They infused quantum dot precursors into microspheres and synthesized quantum dots inside under high temperature and pressure (Figure 3b). In the surface functionalization stage, Zhang et al. [171] used the amphiphilic polymer PSMA to combine quantum dot loading with surface modification. This produced fluorescently encoded microspheres (QD@MPS-PSMA) with carboxyl groups on the surface and quantum dots inside, making them suitable for biosensor applications (Figure 3c). Furthermore, a swelling-silica shell synergistic strategy builds a silica layer on the microsphere surface. This layer helps stably encapsulate quantum dots and allows for surface modification with functional groups for bioconjugation [36,109,154].

**Figure 4:**
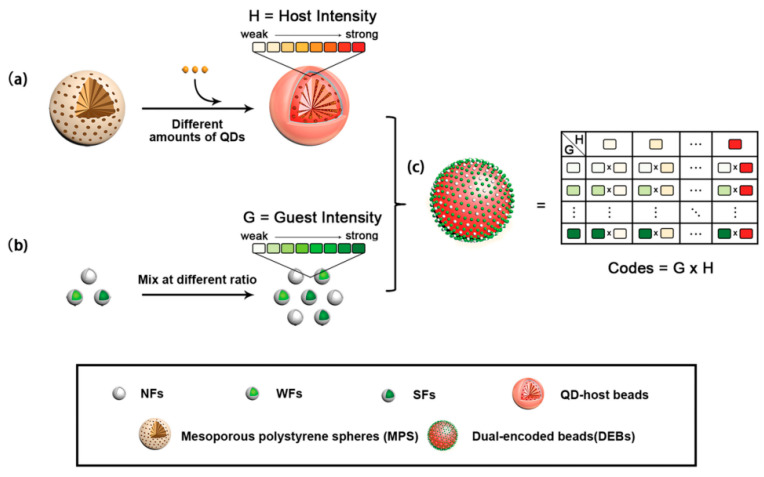
Schematic illustration of the construction strategy for dual-encoding microspheres with host-guest structures [106]. This encoding strategy involves three key steps: (**a**) preparing core–shell QD-host beads by doping mesoporous polystyrene spheres (MPS) with different amounts of QDs600; (**b**) creating guest components through specific mixing ratios of non-fluorescent (NFs), weakly fluorescent (WFs), and strongly fluorescent particles (SFs); and (**c**) chemically conjugating these differentially fluorescent hosts and guests to form DEBs with a vastly expanded encoding capacity.

To address the limitations in encoding capacity and spectral crosstalk caused by high-density loading of fluorophores, layer-by-layer self-assembly provides an effective encoding solution for non-mesoporous microspheres. This method builds multilayer structures by alternately depositing oppositely charged fluorescent nanoparticles and polyelectrolytes onto the microsphere surface. For instance, Lu et al. [172] precisely regulated fluorescence intensity by controlling the number of quantum dot layers assembled. However, conventional LBL technology has certain drawbacks, including strict pH requirements during assembly, signal instability due to easy detachment of fluorescent materials, and a sharp increase in process complexity as more layers are added. To overcome these challenges, the Gu research group [106] proposed an innovative dual-encoding microsphere strategy based on host–guest structures. In this approach, fluorophores are embedded into silica nanospheres as “guest” units, which are then attached to the surface of host microspheres through electrostatic interactions. This design significantly increases encoding capacity while also improving the overall structural stability (Figure 4).

### 3.2. Integration Strategy

**Figure 5:**
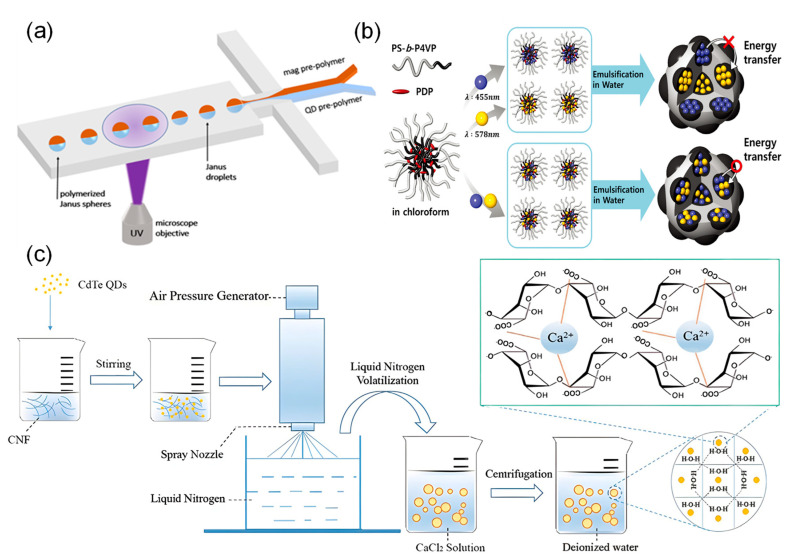
Integration Strategy. (**a**) UV photopolymerization: diagram of a flow-focusing microfluidic device and the formation process of Janus microspheres [151]. (**b**) Emulsification-solvent evaporation method: preparation of block copolymer microspheres containing different-colored quantum dots [179]. (**c**) Spray method: uniform quantum dot encapsulation through atomizing and freezing a quantum dots/cellulose nanofibril mixture, followed by crosslinking and solidification in calcium chloride solution [158].

Unlike methods that apply encoding after microspheres are formed, the integration strategy incorporates fluorescent encoding elements during the microsphere formation process. Key methods include polymerization, emulsification-solvent evaporation, and the spray method. The choice of method depends closely on the properties of the matrix material used. Among available materials, hydrogel microspheres have attracted significant interest in bioassays due to their excellent biocompatibility, three-dimensional network structure, and high probe-loading capacity. Their flexible nature also supports shape-based encodin [35,40,173]. Polymerization is the most common method for producing such microspheres. In this process, surface-modified nanoparticles are mixed with polymer precursors and initiators to form droplets [174,175], which are then polymerized and cured using heat, UV light, or near-infrared radiation [40,151,176,177,178]. As an example, Vaidya et al. [153] used this approach to encapsulate quantum dots inside microspheres but encountered problems with uneven distribution. To improve dispersion uniformity and stability, the Chan group [152] used a technique called concentration-controlled flow focusing. However, this method had difficulty producing microspheres smaller than 4 µm. To achieve better monodispersity and size control, Sha et al. [151] combined UV photopolymerization with microfluidic technology. They successfully prepared two-phase quantum dot-encoded magnetic microspheres with a monodispersity coefficient of only 1.8% (Figure 5a).

In contrast to polymerization, the emulsification-solvent evaporation method allows nanoparticles to be directly mixed and emulsified with polymer precursors in surfactants without prior surface modification. The fluorescent microspheres are then cured after the solvent evaporates [180]. This approach uses evaporation-driven self-assembly to effectively embed fluorescent materials. Ku et al. [179] used this strategy to separate different colored quantum dots inside microspheres, producing encoded microspheres with raspberry-like surface structures. The thicker isolation micelles in this design help reduce fluorescence quenching caused by FRET effects (Figure 5b). To further improve the uniformity and reproducibility of microspheres, researchers have developed microfluidic emulsification [154,155,156] and membrane emulsification-solvent evaporation methods [102,107]. These techniques produce highly uniform droplets using microfluidic channels or Shirasu porous glass membranes, respectively, significantly expanding the controllable size range of microspheres.

The spray method is a high-throughput preparation technique that involves dispersing quantum dots together with matrix materials in a solution and then atomizing the mixture into droplets. These droplets solidify to form encoded microspheres containing quantum dots. Sun et al. [157] first used electrospray technology to prepare quantum dot microspheres with surface carboxyl groups. Separately, Guan et al. [158] introduced an environmentally friendly spray approach. Using water-soluble quantum dots as markers and cellulose nanofibrils as the matrix, they successfully produced dual-color encoded microspheres through a process involving pressure spraying, liquid nitrogen freezing, and calcium ion cross-linking (Figure 5c).

### 3.3. Magnetic Composite Matrix: An Innovative Preparation Strategy for Resolving Fluorescence Quenching

In practical applications, removing matrix interference often requires time-consuming sample pretreatment steps that yield unstable recovery rates [158]. Therefore, developing magnetic suspension biochip technology based on magnetic separation principles, which efficiently enriches target molecules using magnetic beads while simplifying sample pretreatment, holds significant practical value for improving detection efficiency and reliability. The use of superparamagnetic nanoparticles makes it possible to create dual-functional barcodes that combine magnetic separation with optical encoding [37,59,102]. However, the broad optical absorption of these magnetic nanoparticles often leads to fluorescence quenching [102,181]. To address this, researchers have developed various new preparation methods that focus on modifying the matrix materials. Although using near-infrared-emitting quantum dots in magnetic-fluorescent microspheres can reduce such quenching [78,182], this method does not fully resolve the spectral overlap between magnetic nanoparticles and fluorescent materials in the visible range. Zhang et al. [102] used titanium dioxide, which has low absorption in the visible spectrum, to construct Fe_3_O_4_/TiO_2_@QD composite microspheres. By taking advantage of the shielding effect of the dielectric layer, they reduced fluorescence quenching and successfully developed 30 distinct magnetic quantum dot codes. In another approach, Martynenko et al. [144] applied layer-by-layer self-assembly to coat superparamagnetic iron oxide nanoparticles first with a polymer dielectric layer and then with a quantum dot fluorescent shell. Increasing the interlayer distance in this way helps block non-radiative energy transfer. Similarly, Janus particles prepared via emulsification can physically separate fluorescent labels from magnetic nanoparticles, which also greatly suppresses fluorescence quenching [151] (Figure 5a).

### 3.4. Natural Polymers and Molecularly Imprinted Polymers: Green Preparation

Traditional microsphere matrix materials often need complex and environmentally harmful surface modification steps because they lack sufficient functional groups [158,183,184]. In recent years, natural polymers and molecularly imprinted polymers have attracted much interest as new types of matrices due to their sustainable nature and high specificity [158,185]. In the case of natural polymers, Guan et al. [158] used TEMPO-oxidized nanocellulose as a matrix and incorporated dual-color quantum dots into its fiber network through a water-based method. The abundant carboxyl and hydroxyl groups on the nanocellulose surface helped firmly anchor the quantum dots, and this structure greatly improved their fluorescence quantum yield and photostability. As for molecularly imprinted polymers, they offer benefits such as water-based polymerization, high specific surface area, and fast response [186,187,188]. Early studies mainly coated imprinted layers onto quantum dot microspheres to create MIP-modified encoded microspheres [186]. Subsequently, Zhao et al. [187] used functionalized water-compatible imprinted polymers as embedding matrices. This allowed for combination with various quantum dots and solved the compatibility issue between traditional hydrophobic matrices and water-soluble quantum dots. Recently, the same team further developed hollow fluorescent molecularly imprinted microspheres, which shortened the mass transfer paths for quantum dots and other molecules while improving adsorption efficiency [188].

## 4. Signal Amplification

To improve detection sensitivity, signal amplification has become a crucial research focus in encoded microsphere technology. Current approaches mainly include three types: nucleic acid amplification, enzyme catalysis, and nanomaterials. These methods provide crucial support for encoded microsphere platforms to achieve detection at ultra-trace levels.

**Figure 6:**
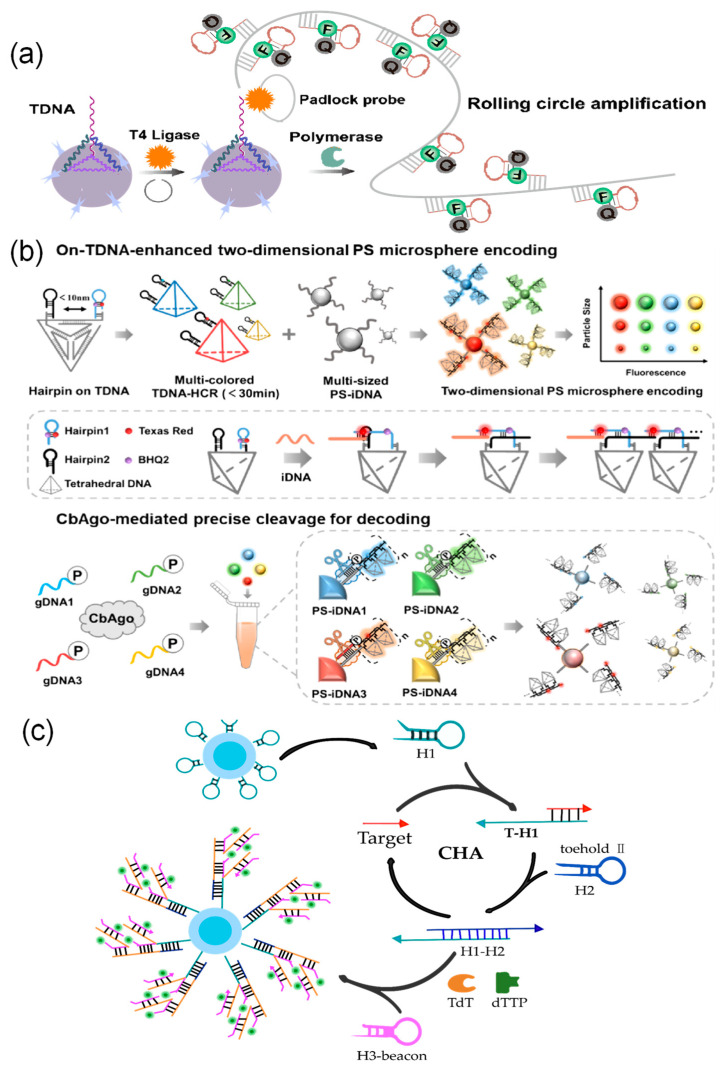
Signal amplification method based on nucleic acid amplification. (**a**) Rolling Circle Amplification (RCA) Technology: In the presence of target DNA, T4 DNA ligase cyclizes probes, followed by DNA polymerase-catalyzed RCA reactions. This generates abundant repetitive DNA sequences on microsphere surfaces, achieving signal amplification. (**b**) Tetrahedral DNA hybridization chain reaction amplification technology [33]: A two-dimensional fluorescently encoded library is constructed by loading target-triggered multicolor DNA nanowires onto microspheres of varying sizes. Subsequently, the CbAgo/gRNA complex programmatically cleaves specific encoded signals, enabling highly specific recognition of multiple targets. (**c**) Catalytically driven hairpin assembly cascade amplification strategy: Catalytically driven hairpin assembly generates TdT primers, which then initiate template-independent DNA polymerization reactions, achieving highly sensitive detection.

Signal amplification methods based on nucleic acid amplification can be divided into two categories based on how they work: target amplification and signal labeling amplification. A comparative overview of the mechanisms and characteristics of these common technologies is provided in Table 3. In the target processing stage, Chan et al. [189] combined recombinase polymerase amplification with quantum dot-encoded microspheres to directly amplify target nucleic acids through enzyme reactions, enabling detection at the femtomolar level with high sensitivity. At the signal labeling stage, several techniques increase the number of probes to achieve signal amplification. Wei et al. [190] used rolling circle amplification to synthesize long DNA chains containing many repeating sequences on microsphere surfaces. This increased fluorescence intensity by about four times and improved sensitivity by two times (Figure 6a). Enzyme-free methods such as hybridization chain reaction and catalytic hairpin assembly can also achieve high sensitivity, detecting targets at femtomolar to picomolar concentrations. These techniques work by triggering hairpin probes to self-assemble into DNA nanostructures or produce cyclic primers when they recognize a target [146,160]. Among these, Wang et al. [33] combined tetrahedral DNA structures with hybridization chain reaction to create a TDNA-HCR system. This system can open multiple hairpin probes at the same time, raising fluorescence intensity by 80% and shortening reaction time by 67% (Figure 6b). Furthermore, catalytic hairpin assembly can work together with terminal deoxynucleotidyl transferase to build cascade amplification systems. For example, Xu et al. [160] used catalytic hairpin assembly to repeatedly release targets and generate primers for terminal deoxynucleotidyl transferase within magnetic quantum dot-encoded microspheres. This started a template-free DNA extension process, leading to cascade amplification as many signal probes attached (Figure 6c).

**Figure 7:**
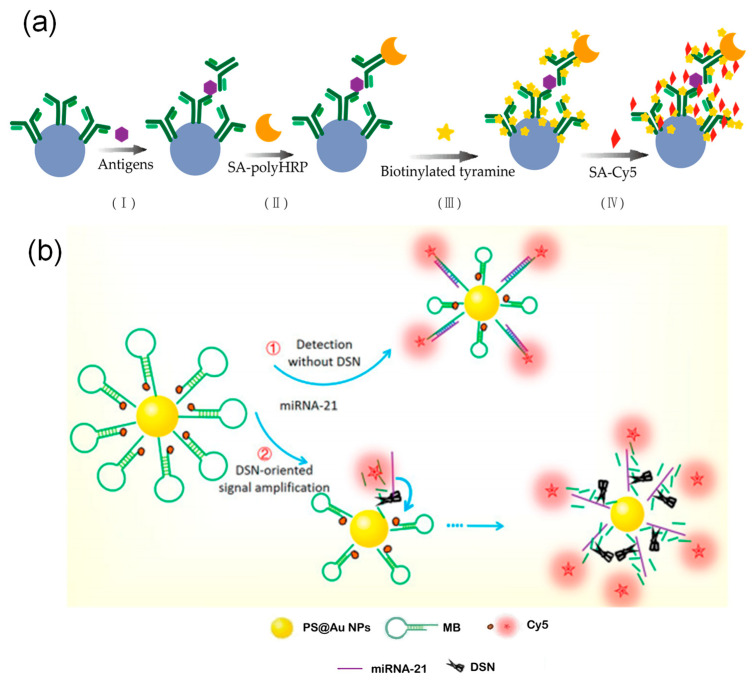
Amplification technologies based on enzyme-catalyzed amplification. (**a**) Tyramide signal amplification: Tyramide signal amplification via combination with multicolor-encoded microspheres. (**b**) Duplex-specific nuclease (DSN) signal amplification: By employing DSN to cleave DNA-RNA hybrids and release fluorophores while recycling miRNA, the detection limit was enhanced by four orders of magnitude [191].

Enzyme-catalyzed amplification is another crucial strategy for improving detection sensitivity. In addition to polymerase-dependent methods such as RPA and RCA, tyramide signal amplification technology enriches signals by using horseradish peroxidase to catalyze the deposition of signal molecules near target sites. Zhang et al. [48] combined this approach with multicolor-encoded microspheres to achieve femtomolar detection limits in tumor marker assays, demonstrating 30 to 15,000 times higher sensitivity than conventional methods (Figure 7a). Furthermore, various enzyme-assisted cyclic cleavage mechanisms are widely used for signal amplification. Zhao et al. [191] applied duplex-specific nuclease to specifically cleave DNA in DNA–RNA hybrid strands, releasing fluorescent groups and intact miRNA. The released miRNA can then re-enter the reaction cycle, improving the detection limit by four orders of magnitude (Figure 7b). Meanwhile, Hu et al. [41] developed a dual-stirring system based on exonuclease I, which releases target bacteria through enzymatic cleavage and triggers multiple rounds of signal tag cycling. When combined with electrochemically encoded microspheres, this system achieved highly sensitive detection at 4 to 7 CFU/mL. In comparison, deoxyribozyme (DNAzyme), as an endogenous DNA catalyst, enables autocatalysis with the help of metal ions and does not require exogenous proteases [192]. Taking advantage of this property, Wang et al. [163] integrated a DNAzyme system with size-encoded microspheres. Target miRNA induces the formation of active DNAzyme structures that cleave substrate strands and restore fluorescent signals. The released miRNA can then repeatedly activate other DNAzymes, resulting in exponential signal amplification.

Nanomaterials enable highly efficient signal amplification through various physical mechanisms. Conjugated polymers, known for their high fluorescence intensity and photostability, can be used to prepare fluorescent microspheres that emit light ten times more intensely than traditional microspheres [193]. In another approach, Sheng et al. [27] used noble metal nanostructures to excite surface plasmon resonance, which enhanced fluorescence intensity by 180 times and lowered the detection limit by about 1000 times. In addition, Duan et al. [107] achieved detection sensitivity at the picogram per milliliter level by using quantum dot nanobeads, where each bead encapsulates thousands of quantum dots, realizing a high loading strategy.

The aforementioned signal amplification strategy significantly enhances detection sensitivity. However, since background signals and non-specific interactions undergo equivalent amplification effects, this may generate false-positive results. Gaining a deep understanding and controlling the sources of non-specific signals is crucial for achieving high-precision detection. In nucleic acid amplification technologies, false positive signals primarily originate from non-specific amplification and primer dimer formation. For instance, RCA carries the risk of non-specific amplification due to imprecise matching, while HCR may accumulate background signals because the hairpin probes it employs can spontaneously unfold and hybridize in solution [194]. Additionally, aerosol-borne contamination of target nucleic acids can occur in microsphere-based solid-phase reaction systems, leading to false amplification. False positive signals in enzyme-catalyzed amplification may originate from fluctuations in enzyme activity itself and non-specific cleavage by enzymes generating false signals [194]. In nanomaterial-mediated signal amplification, the surface of encoded microspheres may non-specifically bind to other components through hydrophobic interactions, electrostatic adsorption, etc. [195]. Mitigating these false-positive risks can be achieved by integrating several practical steps. We can begin by pre-treating encoded microsphere carriers with blocking agents like BSA to reduce non-specific protein adsorption. Furthermore, meticulously designing hairpin probes and optimizing thermal protocols help minimize off-target hybridization and primer-dimer artifacts. Crucially, incorporating physical isolation measures into the experimental setup is necessary to guard against aerosol-borne contamination.

## 5. Technical Application Platform of Encoded Microsphere Suspension Arrays for Mycotoxin Detection

Encoded microsphere suspension array technology has become a key method for high-throughput and highly sensitive detection of mycotoxins, as it allows for the simultaneous analysis of multiple markers in a single sample. In contrast, traditional chromatographic techniques such as ultra-high-performance liquid chromatography–tandem mass spectrometry [196] and gas chromatography–tandem mass spectrometry [197] are limited by complex sample preparation, low throughput, and long processing times. The suspension array technology integrates multiple detection platforms and shows significant advantages in improving detection performance and application flexibility. Currently, instruments including flow cytometers, fluorescence microscopes, micro-resistance platforms, and smartphones are widely used to meet detection requirements in various scenarios [23,66,156,173,198,199].

Among these methods, flow cytometry has become widely used in encoded microsphere suspension arrays because of its high sensitivity, fast response, and strong reliability. It reads fluorescence signals from microspheres one by one, enabling target detection with only microliter volumes of reagents [200]. The data collection ability of this technology comes from the efficient binding between the high-surface-area microspheres and probe molecules, allowing multiple targets to be analyzed at the same time [201]. Zhang et al. [198] added upconversion nanoparticles and magnetic nanoparticles into carboxylated porous polystyrene microspheres. Using dual laser excitation at 980 nm and 488 nm in a flow cytometer, they separately collected encoding and detection signals. This method reached a detection limit as low as 9 pg/mL for target toxins, showing a 100-fold improvement in sensitivity over conventional HPLC. It also provided high accuracy and reproducibility in spiked corn recovery tests.

In optical imaging platforms, fluorescence microscopes use specific wavelengths of light to activate fluorescent labels on encoded microspheres. The resulting fluorescence signals pass through filters and are captured by detectors, then analyzed by image processing software to identify microsphere types and measure target concentrations [202]. Yang et al. [66] created a dual-channel detection system using a portable fluorescence microscope. This system employed blue and red upconversion nanoparticles to encode microspheres for specifically labeling ochratoxin A and zearalenone, respectively. The setup used a 980 nm laser to excite the background-free encoded signal, while blue light excited the PE-labeled secondary antibody to produce the detection signal. This approach achieved highly sensitive detection of ochratoxin A and zearalenone, with detection limits of 0.35 ng/mL and 0.41 ng/mL, respectively, and R^2^ values greater than 0.97. The method also showed high accuracy and reproducibility in spiked corn experiments. Combined with image analysis algorithms, this system is suitable for rapid on-site screening in settings with limited resources.

Microporous resistance counting technology is widely used for analyzing particle number and size due to its high sensitivity, strong precision, and low cost [203]. Li et al. [23] developed a microporous resistance counting platform using polystyrene microspheres of different sizes (3 μm, 4 μm, and 6 μm) for encoding, combined with CbAgo-based decoding, to simultaneously detect aflatoxin B1, ochratoxin A, and deoxynivalenol. This technology improves both detection sensitivity and multiplexing capability by distinguishing microspheres based on their size. The study also introduced an innovative “lollipop”-shaped probe structure. The polystyrene head of this probe physically adsorbs antibodies, allowing for efficient separation of complexes and reducing matrix interference with only 35 s of vortex washing. This platform achieves sensitivity at the picogram per milliliter level across four orders of magnitude. It provides a practical solution for rapid mycotoxin detection that meets the need for high sensitivity, multiplex analysis, and low cost.

With the growing demand for portable testing, smartphone-based image processing combined with point-of-care devices offers new solutions for on-site mycotoxin detection. Ji et al. [173] used stop-flow lithography to produce shape-encoded hydrogel microspheres in microfluidic channels, where squares represented ochratoxin A and circles represented aflatoxin B1. A 488 nm laser excited PE-labeled complementary strands to produce fluorescent signals. A smartphone camera captured particle images and identified toxin types based on shape, while fluorescence intensity allowed for quantitative analysis. Their integrated image algorithm completed shape recognition and digital analysis of fluorescent regions within 10 s, enabling simultaneous detection of both toxins. The detection ranges were 0.1–200 ng/mL for aflatoxin B1 and 0.1–500 ng/mL for ochratoxin A, with spiked corn recovery rates of 81.5–121%. The entire process took under 105 min, showing high throughput and practicality. In another study, Yang et al. [199] prepared green, blue, and red upconversion nanoparticles doped with different rare-earth elements as encoding signals. Using 980 nm and 488 nm lasers to excite encoding and reporter fluorescence, respectively, a smartphone with a custom application completed multiplex mycotoxin detection within 1 min, achieving a detection limit of 1 ng.

## 6. Conclusions and Perspectives

This review systematically examines suspension array technology based on encoded microspheres, with a focus on core aspects such as encoding strategies, microsphere construction, and signal amplification, along with its recent applications in multiplex detection of mycotoxins. It thoroughly explores the basic principles and development path of each technique. In recent years, advances in nanotechnology have driven significant progress in this field. However, we must not overlook the fact that fluorescent-encoded microspheres successfully synthesized in the laboratory are difficult to produce on a large scale. Moreover, the high-precision manual preparation process incurs significant costs. Therefore, we urgently need to develop production technologies capable of achieving batch production with high consistency. Additionally, the long-term stability issues encountered by fluorescent-encoded microspheres in practical applications necessitate the development of more inorganic encapsulation techniques. For instance, inorganic materials such as silica can be utilized to form protective shells on the surface of the microspheres.

Looking ahead, the technology still has great potential for further improvement. A key trend is the intelligent development of encoding and decoding processes. By incorporating artificial intelligence algorithms, it becomes possible not only to accurately decode complex signals but also to design optimal encoding strategies, helping to overcome capacity limitations [165,204]. In microsphere construction, choosing biocompatible natural polymers and low-toxicity quantum dots, along with advanced fabrication techniques such as 3D printing, will allow for more precise control over microsphere structure and the distribution of encoding elements [205]. At the same time, reducing signal interference requires a comprehensive strategy. Magnetic encoding can improve matrix separation, near-infrared probes can lower background fluorescence, molecularly imprinted polymers can increase recognition specificity, and smart algorithms can identify and exclude microspheres affected by non-specific binding. These approaches will significantly improve detection accuracy and reliability in the future. In terms of signal amplification, programmable DNA nanotechnology can be used to build dynamic and efficient cascade amplification systems, which are expected to further lower detection limits. Ultimately, future detection platforms will not be limited to reading only optical signals from microspheres. They will also be able to collect multiple physical and chemical parameters, including size, composition, and motion behavior. Together with the use of portable devices and smartphones, these systems will become powerful tools well suited for rapid on-site testing.

In summary, through the integration of materials science, nanotechnology, optics, and information technology, encoded microsphere suspension array technology is expected to develop into a smarter, more sensitive, and more practical analytical platform. It will not only be valuable to food safety monitoring but also has significant potential in other fields such as precision medicine and environmental monitoring.

## Figures and Tables

**Table 1:** Summary of multiple fluorescent encoding strategies.

Encoding Dimension	Encoding Methods	Barcode Number	Decoding Method	Advantages	Disadvantages or Problems to be Solved
Wavelength and intensity	Organic dyes	500 [46]	FCM	Low cost and good stability in solvents [47,48]	Limited variety of available dyes; self-quenching effect; poor light stability; requires multiple excitation wavelengths [28,49]
Wavelength and intensity	QDs	144 [22]	FCM	Narrow emission peaks; high quantum yield; single-wavelength excitation; tunable emission wavelength [50,51,52,53,54,55,56,57]	Presence of FRET or absorption crosstalk; potential heavy metal toxicity [58,59,60,61,62,63,64]
Wavelength and intensity	UCNPs	10 [65]	fluorescence microscope [66]	Minimal signal crosstalk; narrow emission peaks; low biotoxicity and high stability [67,68,69,70,71]	Limited compatibility with standard equipment and lack of specialized excitation sources [72]
Wavelength and intensity	AIEgens	30 [73]	FCM [73,74]	Resistant to aggregation-caused quenching; low biological toxicity [75,76,77]	Broad transmission peaks, usually allowing for only single-wavelength encoding [64,78]
Wavelength and intensity	FP	223 [28]	FCM [28]	High biocompatibility; environmentally friendly [28]	Inconsistent quantum yield; complex preparation process [28]
Optical structures	SERS	4 [79] 26 [80]	Confocal Raman Microscope [79,80]	Resistant to photobleaching [81,82]; high sensitivity [83]; stable signals and strong multiplexing capability [84]	Uneven heat distribution; low stability when bound to metals; high equipment cost [79,85,86,87]
Optical structures	Photonic Crystal	3 [88]	fluorescence microscope [88]	Resistant to photobleaching; excitation by white light; low cost [88,89]	Limited encoding capacity; complex assembly procedure [88,90]
Temporal and spatial characteristics	Life-time	9 [34] 6 [91]	TCSPC- Fluorescence Microscope System [34]; Time-Gated Imaging System [91]	Lifetime can be adjusted via FRET; suitable for trace-level detection [34,60,92]	Encoding capacity limited by lifetime resolution; complex preparation; signals prone to drift; high system cost [34,60,93]
Temporal and spatial characteristics	FA	8 [94]	LSCM [94]	Avoids spectral crosstalk [94]	Low stability; susceptible to interference from other molecules and environmental factors [95,96]

FCM: Flow Cytometry; QDs: Quantum Dots; FRET: Förster Resonance Energy Transfer; UCNPs: Upconversion Nanoparticles; AIEgens: Aggregation-Induced Emission Luminogens; FP: Fluorescent Protein; SERS: Surface-Enhanced Raman Scattering; FA: Fluorescence anisotropy; LSCM: Laser Scanning Confocal Microscope.

**Table 2:** Comparison of Incorporation Strategies and Technical Characteristics for Different Preparation Methods of Encoded Microspheres.

Preparation Method	Applicable Substrate	Technical Principles	Advantages	Disadvantages or Problems to be Solved
swelling method	PS, SiO_2_	Porous microspheres adsorb dyes after swelling and are embedded after solvent removal	Simple to operate and low cost	Polar solvents may cause fluorescence quenching; multiple processing steps needed; limited encoding capacity and prone to crosstalk [148,149]
LBL	PS, SiO_2_, Magnetic microspheres	Alternating deposition of fluorescent materials and polyelectrolytes using electrostatic or hydrogen bonding to build multilayer films	Allows for use of multiple dyes while reducing crosstalk	Low signal stability due to easy detachment of layers; complex and time-consuming process; poor reproducibility [150];
Polymerization	Hydrogel, MIPs	Surface-modified fluorescent nanoparticles are copolymerized with polymer precursors, followed by crosslinking and curing	Uniform particle distribution; high stability; suitable for large-scale production [151,152]	Poor compatibility can lead to uneven distribution and increased risk of fluorescence quenching [153]
Emulsification-solvent evaporation	PS, Magnetic microspheres	Fluorescent nanoparticles are emulsified with polymer precursors without surface modification; microspheres form as solvent evaporates	Combines self-assembly for high encapsulation efficiency; easily produces uniform microspheres at large scale [102,107,154,155,156]	Relatively high cost; organic solvent residues may cause biotoxicity [102,107]
Spray method	natural polymers	A solution containing dyes is sprayed and rapidly forms solid microspheres through drying or crosslinking	Excellent particle uniformity; one-step formation; highly efficient process [157]	High equipment cost; uneven dye distribution; electrospray sensitive to voltage changes [157]; microspheres made by green spray tend to be larger [158]

PS: Polystyrene; LBL: Layer-by-Layer; Molecularly; MIPs: Imprinted Polymers.

**Table 3:** Comparison of Signal Amplification Technologies Based on Nucleic Acid Amplification.

Technology	Working Principle	Enzyme	Amplification Mechanism	Advantages
RPA	target amplification	Enzyme-dependent	Directly amplifies target nucleic acid sequences through enzymatic reactions under constant temperature	Fast (10–15 min); no need for temperature control equipment; suitable for point-of-care testing [189]
RCA	signal labeling amplification	Enzyme-dependent	Produces long DNA strands with many repeating sequences on the microsphere surface via rolling circle amplification	Very high sensitivity; large encoding capacity; suitable for single-molecule detection [190]
HCR	signal labeling amplification	Enzyme-independent	Forms double-stranded DNA products through strand displacement reactions using hairpin probes	Low cost [33,146]
CHA	signal labeling amplification	Enzyme-independent	Generates DNA primers and signal structures through catalytic hairpin assembly cycles	High sensitivity; reaction completes in under 30 min; can be combined with TdT enzyme for cascade amplification [160]

RPA: Recombinase Polymerase Amplification; RCA: Rolling Circle Amplification; HCR: Hybridization Chain Reaction; CHA: Catalytic Hairpin Assembly.

## Data Availability

No new data were created or analyzed in this study. Data sharing is not applicable to this article.
